# Tualang honey supplementation as cognitive enhancer in patients with schizophrenia

**DOI:** 10.1016/j.heliyon.2020.e03948

**Published:** 2020-05-12

**Authors:** Rosliza Yahaya, Mohd Nizam Zahary, Zahiruddin Othman, Rusli Ismail, Nik Ahmad Shaiffudin Nik Him, Aniza Abd Aziz, Rahima Dahlan, Azizul Fadzli Jusoh

**Affiliations:** a Faculty of Medicine, Medical Campus, Universiti Sultan Zainal Abidin, Jalan Sultan Mahmud, 20400 Kuala Terengganu, Terengganu, Malaysia; b Faculty of Health Sciences, Gong Badak Campus, Universiti Sultan Zainal Abidin, 21300 Kuala Nerus, Terengganu, Malaysia; c School of Medical Sciences, Health Campus, Universiti Sains Malaysia, 16150 Kota Bharu, Kelantan, Malaysia; d Department of Psychiatry, Faculty of Medicine and Health Sciences, Universiti Putra Malaysia, 43400 Serdang, Selangor, Malaysia

**Keywords:** Neuroscience, Psychiatry, Biological psychiatry, Clinical psychology, Alternative medicine, Clinical research, Schizophrenia, Cognitive enhancer, Honey, AVLT

## Abstract

**Introduction:**

Schizophrenia is a chronic mental illness with clusters of symptoms, including cognitive impairment. This study aimed to explore the effect of Tualang Honey (TH) on cognitive domains, especially as it pertained to the verbal memory of schizophrenia patients.

**Method:**

This was a cross-sectional study involved 80 individuals, diagnosed with schizophrenia. The Malay Version Auditory Verbal Learning Test (MVAVLT) was used. Data were analysed using SPSS 20.0 software. Intention to treat analysis was applied.

**Result:**

A comparison of the total learning score at eight weeks between the two groups based on time effect and time-treatment interaction favoured TH group.

**Conclusion:**

This study concludes that by supplementing schizophrenia patients with 8-week of TH did improve total learning performance across domains in the immediate memory among patients with schizophrenia.

## 1 Introduction

Schizophrenia is a chronic mental illness with major clusters of symptoms, including positive symptoms, negative symptoms and cognitive impairment. It is debilitating and common. It occurs in approximately 1% or nearly 21 millions of world population (WHO, 2018). Locally, the National Mental Health Registry (NMHR) at the Ministry of Health Malaysia reported an incidence rate of between 7.3 to 43.0 per 100 000 population (Malaysia, 2005). A weakening in at least one of the cognitive function domain occurred in 80% of schizophrenia patients (R. S. E. Keefe and Fenton, 2007) and cognitive impairment is a core feature. Cognitive impairment is both predictive for functional outcomes and a treatment target (Green et al., 2004). Cognitive dysfunction can indeed be present before the onset of psychotic symptoms, and after the first psychosis, it either remains at decreased levels or declines during the illness (Kahn and Keefe, 2013). The most impaired cognitive domain is verbal memory (Cirillo and Seidman, 2003) which is associated with a poor functional outcome (Barch and Ceaser, 2012) and impaired executive function (Abdel-Hamid et al., 2009). The pathophysiology of cognitive dysfunctions in schizophrenia is complex and involves many different neurotransmission systems, including the glutamatergic and cholinergic system (R. S. Keefe et al., 2013). Cognitive enhancers have been a primary focus of research in the last decades, and have shown promising but inconclusive results (Sinkeviciute et al., 2018).

Honey is a sweet, viscous food substance produced by bees and some related insects. In myths and folk medicine, honey has been used to treat various ailments by ancient peoples and in Ayurveda and traditional Chinese medicine. More recently, extensive studies have been conducted on the application of honey in modern medicine. Locally, the Tualang honey (TH), in particular, is used in diverse medical conditions based on its many different properties. TH contains polyphenols with antioxidant (Moniruzzaman et al., 2013), antibacterial (Nasir et al., 2010), and antiproliferative (Nurul Syazana, Halim, Gan and Shamsuddin, 2011) activities. It promotes wound healing (Khoo et al., 2010). A recent study also showed that TH ameliorated impaired immediate memory among postmenopausal women (Othman et al., 2011) and reduced anxiety in rats (B. Al-Rahbi et al., 2013). It is hypothesised that TH has a role in improving learning and memory via (i) a reduction of oxidative stress, (ii) an enhancement of the cholinergic system, (iii) an induction of the synthesis and secretion of brain-derived neurotrophic factor (BDNF) and (iv) an augmentation of anti-inflammatory properties. Therefore, this study aimed to explore the effect of TH on cognitive domains, especially as it pertained to the verbal memory of schizophrenia patients.

## 2 Materials and methods

### 2.1 Study subjects

The study involved 80 individuals, aged 18–55 years, diagnosed with schizophrenia who were attending the psychiatry outpatient clinic at Hospital Universiti Sains Malaysia (HUSM) and fulfilled the inclusion and exclusion study criteria (Table 1) were consecutively invited to enrol in this study.

**Table 1:** Inclusion and exclusion study criteria.

Inclusion Criterias	Exclusion Criterias
• Schizophrenia patient according to the (DSM-5)	• Cognitive disorder such as delirium and dementia
• BPRS score <30	• Substance intoxication or withdrawal
• Able to speak understand Malay language	• Intellectual disabilities
	• Neurocognitive impairment
	• Medical condition that affected with intervention substances such as diabetes mellitus or acromegaly.
	• Severe communication problems such as deafness and mute
	• Regular benzodiazepine or anticholinergic medications

Diagnostic and Statistical Manual of Mental Disorders, 5th edition (DSM-5), Brief Psychiatric Rating Scale (BPRS).

Sociodemographic data and personal details of the patients including age, gender, ethnicity, marital and employment status, educational level, religion, number of hospitalisation, the overall duration of illness, treatment and type of pharmacotherapy were recorded. Eligible patients were randomly assigned to control and intervention groups by block allocation randomisation.

The TH was obtained from the Federal Agriculture Marketing Authority (FAMA) in sachet form. Each sachet consisted of 20 g TH. FAMA did the processing steps from harvesting, sterilising and packaging based on its standard protocol. The physiochemical and safety characteristic were analysed and validated by a GMP certified laboratory. Forty "control" subjects continued their prescribed medical treatment without honey, and another 40 were maintained on their medical treatment to which was added eight-week supplies of TH to be consumed at 20gm daily, every morning at 8.00 am. The honey was supplied in sachets every fortnightly. Empty sachets were exchanged with new sachets, and their daily honey intake was monitored by relatives to ensure compliance.

Ethical approval was obtained from the Human Research and Ethics Committee, School of Medical Sciences, Universiti Sains Malaysia (HREC), study protocol code USM/JEPeM/140355. Informed consent was obtained from all study subjects before the study commenced.

### 2.2 Research tools

The Malay Version of Auditory Verbal Learning Test (MVAVLT) (Ruzita Jamaluddin et al., 2009) was used to evaluate short-term memory, to retain and recall information after some impediment. The tool was translated from World Health Organization/the University of California, Los Angeles (WHO/UCLA) version of the Auditory Verbal Learning Test (Maj et al., 1993; Rey, 1958) and yielding a Cronbach alfa value of 0.84, and test-retest correlations ranged from 0.24 to 0.84 (Ruzita Jamaluddin et al., 2009). There were two distinct series of items to be recognised comprising of List A, List B, together with a Recognition List. Each series consisted of 15 concrete nouns which are commonly used in local conversation across all ages group.

The test affords the ability of a person to encode, consolidate, store, and retrieve verbal information, and is usually applied using a five-trial presentation of a 15-noun word list (list A) (presentation rate of one word per second), a single presentation of a 15-noun word interference list (list B), two post-interference recall trials—one immediate, one delayed (ranging from 20 to 45 min, most commonly around 30 min) and a recognition trial of 50 words containing the target words of lists A and B and 20 distractor words phonetically or semantically similar to those in lists A and B (M.D. Lezak, 2004; Schmidt, 1996). The listed words were documented before the study to ensure consistency.

A trained enumerator or the researcher conducted the test. Inter-rater reliability analysis showed high agreement between the ratters in the first exposure of the evaluation with 97.3%.

BPRS (Overall and Gorham, 1962) was also applied to assess psychotic symptoms. BPRS consisted of 18-symptoms constructs that took 20–30 min for the assessment and scoring. Evaluations were administered at week 0 (pre-) and repeated at week 8 (post-intervention) of the two-month follow-up period.

### 2.3 Data analysis

Data were analysed using SPSS 20.0 software (supplier). Intention to treat analysis was applied. Results are presented as means and standard deviations (SD), or medians and interquartile ranges (IQR), or as percentages (%), as appropriate. Univariate analyses were performed to compare baseline characteristics between control and intervention groups. Independent t-test was applied for numerical variables. Pearson chi-square test was used for categorical variables. Repeated measures analysis of covariance (RmANCOVA) was applied to analyse dependent variables, total learning and delayed recall scores of MVAVLT with two levels of measurements: before intervention (pre) at week 0 and after intervention (post) at week 8. All statistical tests were two-sided, and P < 0.05 was considered to indicate statistical significance.

## 3 Results

**Figure 1:**
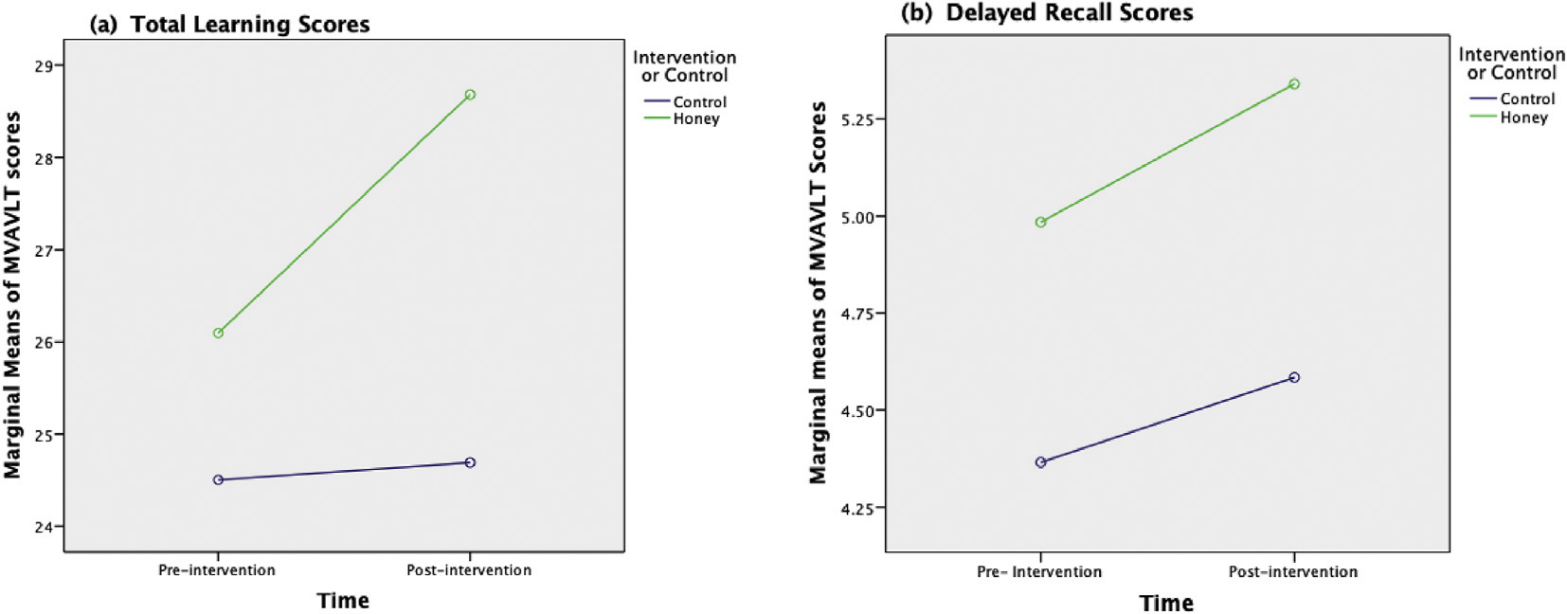
(a): Total learning score and (b): Delayed recall scores of MVAVLT performance in control group (n = 40), and honey group (n = 40) at pre-intervention and intervention. The differences between pre-intervention and post-intervention scores for each group were analysed using RM ANCOVA. Covariates appearing in the model are evaluated at the following values: Age in years = 30.98, Education in years = 12.66, Overall duration of illness in months = 107.68. MVALVLT, Malay version of the Auditory Verbal Learning Test, RM ANCOVA, Repeated measure analysis of covariance.

Eighty subjects met the criteria and were consented and randomised into control and TH groups. There were no significant differences in sociodemographic and clinical characteristic between the groups at baseline (Table 1) (see Figure 1).

A comparison of the total learning score between the two groups based on time (time effect) favoured TH group. The TH group demonstrated a significantly higher mean total score compared to the control group at eight weeks (2.40; 95%CI:0.53,4.27 vs 0.38; 95%CI:1.00,1.75 (Table 2).

**Table 2:** Socio-demographic and clinical characteristics of subjects (n = 80).

Characteristic	TH group (n = 40)		Control group (n = 40)		p-value
	Mean (SD)	Frequency (%)	Mean (SD)	Frequency (%)	
Age in years	31.43 (8.68)^a^		30.53 (7.89)		0.629^c^
Age at first treatment (years)	22.05 (7.55)^a^		22.70 (6.82)		0.688^c^

Sex					0.370^b^

Male		21 (55.3)		17 (44.7)	
Female		19 (45.2)		23 (54.8)	

Marital status					0.533^b^

Single		12 (57.1)		9 (42.9)	
Married		3 (60.0)		2 (40.0)	
Divorced/Separated		2 (100.0)		0 (0.0)	

Employment					0.937^b^

Unemployed		19 (46.3)		22 (53.7)	
Employed		21 (55.3)		17 (44.7)	
Educational (years)	12.70 (2.52)		12.63 (2.20)		0.888^c^
Duration of illness (months)	112.65 (86.82)^a^		92 (66.30)		0.235^c^
Duration of treatment (months)	119.55 (86.88)^a^		95.80 (66.18)		0.173^c^

Admission					0.237^b^

Never been admitted		11 (40.7)		16 (59.3)	
At least admitted once		29 (54.7)		24 (45.3)	

Antipsychotic					0.260^b^

Atypical		22 (46.8)		25 (53.2)	
Typical		15 (62.5)		9 (37.5)	
Both		3 (33.3)		6 (66.7)	

a Median (IQR).

b Chi-square test.

c Independent t test.

Similarly, a significantly higher mean score was observed compared with the control group (time-treatment interaction) 28.86; 95%CI: 25.13, 32.23 vs 24.69; 95%CI: 21.14, 28.24 (F = 4.46, p = 0.038) (Table 3).

**Table 3:** Comparison of MVALT score Trials within each treatment group based on time (Time effect).

Comparison	Control		Honey	
	MD (95% CI)	p- value	MD (95% CI)	p- value
Total learning				

Pre-Post Total (A1 + A2 + A3 + A4 + A5)	0.38 (1.00,1.75)	0.584	2.40 (0.53, 4.27)	0.013∗

Delayed recall				

Pre- Post A7	0.25 (0.07,0.57)	0.122	0.33 (0.05,0.70)	0.085

Repeated measure ANCOVA within-group analysis was applied followed by pairwise comparison with confidence interval adjustment (Bonferroni correction), Binary covariate (age, education duration and illness duration) was controlled by using repeated measure ANCOVA.

MD = mean difference.

Otherwise, there was no interaction between the groups (Table 4) (see Table 5).

**Table 4:** Comparison of MVALT score Trials among two treatment group based on time (Time treatment interaction).

Trials	Time	Treatment Group	Mean Score	95% CI	F- stat (df)	p-value
Total Learning scores						

(A1 + A2 + A3 + A4 + A5)	Pre-treatment	Control	24.50	21.42, 27.59	4.46 (1)	0.038∗
		Honey	26.10	23.01, 29.18		
	Post-treatment	Control	24.69	21.14, 28.24		
		Honey	28.68	25.13, 32.23		

Delayed Recall scores						

A7	Pre-treatment	Control	4.37	3.47, 5.26	0.31 (1)	0.582
		Honey	4.98	4.09, 5.88		
	Post-treatment	Control	4.58	3.68, 5.49		
		Honey	5.34	4.44, 6.25		

A6 scores do not contribute to outcome interpretation.

**Table 5:** Mean difference of MVALT score trials among two (between-group).

Trials	Comparison	Mean Score difference (95% CI)	F-stat (df)	P-value
Total learning				

A1+A2+A3+A4+A5	Control- Honey	2.79 (1.80,17.38)	1.46 (1)	0.230

Delayed Recall				

A7	Control- Honey	0.69 (0.571,1.95)	1.19 (1)	0.280

A6 scores do not contribute to outcome interpretation.

Repeated measure ANCOVA between-group analysis was applied, followed by pairwise comparison.

Binary covariate (age, education duration and illness duration) was controlled by using repeated measure ANCOVA.

Level of significance was set at 0.05 (two-tailed).

Repeated measure ANCOVA between-group analysis with regard to time was applied. Binary covariate (age, education duration and illness duration) was controlled by using repeated measure ANCOVA. Assumption normality, homogeneity of variances, compound symmetry and homogeneity of regression were checked and were fulfilled.

## 4 Discussions

In recent years, there has been renewed interest in the use of natural products to prevent and treat diseases and honey is no exception, especially with the many good testimonies of its usefulness. Its usefulness in difficult-to-treat diseases like schizophrenia would, therefore, be welcome, especially with laboratory and clinical evidence. This study was prompted by observations that increased oxidative stress was associated with a decline in executive function in healthy individuals, Alzheimer's patients and postmenopausal women (Doshi and Agarwal, 2013; Hajjar et al., 2018; Hatanaka et al., 2015). Indeed, there has been a growing interest among researchers on antioxidants (Moniruzzaman et al., 2013; Nasir et al., 2010; Nguyen et al., 2019) in many kinds in health and diseases. In the study of postmenopausal women, TH was associated with a reduction in oxidative stress as evidenced by an elevation of plasma antioxidant activities and decreased plasma lipid peroxidation level (Shafin et al., 2014). TH consumption enhances defence against oxidative stress and attenuates free radical-mediated molecular destruction. This mechanism leads to improved arrangement and number of Nissl positive cells in the mPFC and hippocampal neurons (Badria al-rahbi, Zakaria, Othman, Hassan and Ahmad, 2014).

Our results showed that TH improved WM in the studied schizophrenic subjects. There were significant differences in learning scores according to time effect and time-treatment interaction when controlled for covariates such as age, education and duration of illness between the intervention group and the control group. TH supplementation for eight weeks was associated with improvement in the total learning (immediate memory/STM) and but not with delayed recall (LTM) in our subjects, findings similar to studies among postmenopausal and dementia patients (Al-Himyari, 2009; Othman et al., 2011).

Performance on WM tasks depends on dopamine function in the dorsolateral prefrontal cortex with the cholinergic system exerting a neuromodulatory influence on glutamatergic and GABAergic networks (Arnsten et al., 2012). The cholinergic system in the nervous system is critical for memory formation (Deiana et al., 2011). This is evidenced by the shrinkage and degeneration of basal forebrain neurons alongside the reduction of cholinergic markers in cortical and hippocampal targets in patients with Alzheimer's disease (Schliebs and Arendt, 2011). In a study by Al-Himyari (2009), honey was found to be protective against dementia (Al-Himyari, 2009). Furthermore, an animal study showed increased acetylcholine (ACh) and reduced acetylcholinesterase (AChE) concentrations in stressed ovariectomised rats fed with TH. TH supplementation was shown to exert beneficial effects on the medial prefrontal cortex (mPFC) and the cholinergic system comparable to the effect of 17β-oestradiol treatment (Badria al-rahbi et al., 2014). Since TH is rich in flavonoids and contains the highest total phenolic content compared to other types of honey, TH may exert its positive effects through both its antioxidant properties as well as through its effects on choline and ACh contents (Khalil et al., 2011).

It is interesting also to postulate that TH antioxidant, and anti-inflammatory properties (Moniruzzaman et al., 2013) together with its effects on BDNF probably also played some roles, probably linked to cholinergic abnormalities in schizophrenia (Sinkeviciute et al., 2018). TH is a phytoestrogen-rich in flavonoids, substances shown to increase hippocampal BDNF levels. BDNF belongs to the neurotrophin family that is secreted in the highest amount in the brain. It plays a vital role in long-term hippocampal potentiation linked to learning and memory (Leal et al., 2015). Siuda et al. found that low serum BDNF was associated with cognitive impairment and neurodegenerative disease (Siuda et al., 2017). TH or E2-treated stressed ovariectomised rats also showed reduced serum adrenocorticotropic hormone and cortisone levels and depressive-like behaviour in stressed ovariectomised rats (B. Al-Rahbi et al., 2013).

Memory can be categorised based on temporal durabilities such as long-term or short-term memory, or according to the content of information and circuitry such as declarative (explicit) or non-declarative (implicit). Short-term memory (STM) refers to the capability of retaining quick information over a specified period. Working memory (WM) refers to the ability to coordinate or manoeuvre and retain the information (transiently stored) during cognitive activities (Camina and Guell, 2017). Cognitive impairment in WM was demonstrated in schizophrenia patients across all verbal memory, visuospatial and executive working memory (Forbes et al., 2009).

This study is being carried out in light of the promising results on TH supplementation as a cognitive enhancer. However, it is also pertinent to highlight some limitations in this study. Firstly, the sample size is considered small. It is imperative to design the research with a larger sample size if we are dealing with the outcome measures such as a cognitive performance from this particular study population. Secondly, a brief intervention with a total duration of the 8-week supplementation period may influence the result of the study. Hence, future long-term study with larger sample size is needed to accurately investigate the possible effects of Tualang honey as cognitive enhancer among schizophrenia patients.

## 5 Conclusion

This study concludes that by supplementing schizophrenia patients with 8-week of TH did improve total learning performance across domains in the immediate memory but not in long-term memory among patients with schizophrenia. It showed a promising role of TH which are a natural substance, readily available and cost-effective as a cognitive enhancer in a schizophrenic patient. The future direction of the study should address on other cognitive domains, preferably with longer duration of intervention and larger sample size.

## Declarations

### Author contribution statement

Rosliza Yahaya: Performed the experiments; Wrote the paper.

Mohd Nizam Zahary, Rusli Ismail: Conceived and designed the experiments; Contributed reagents, materials, analysis tools or data.

Zahiruddin Othman: Conceived and designed the experiments; Performed the experiments.

Nik Ahmad Shaiffudin Nik Him, Rahima Dahlan: Performed the experiments; Analyzed and interpreted the data.

Aniza Abd Aziz: Conceived and designed the experiments; Analyzed and interpreted the data.

Azizul Fadzli Jusoh: Analyzed and interpreted the data; Wrote the paper.

### Funding statement

This work was supported by the Ministry of Higher Education for financial aid (FRGS/1/2017/SKK03/UNISZA/03/1).

### Competing interest statement

The authors declare no conflict of interest.

### Additional information

No additional information is available for this paper.

## References

[bib1] Abdel-Hamid M., Lehmkamper C., Sonntag C., Juckel G., Daum I., Brune M. (2009). Theory of mind in schizophrenia: the role of clinical symptomatology and neurocognition in understanding other people's thoughts and intentions. Psychiatr. Res..

[bib2] Al-Himyari F.A. (2009). The use of honey as a natural preventive therapy of cognitive decline and dementia in the middle east. Alzheimer's Dementia.

[bib3] al-rahbi B., Zakaria R., Othman Z., Hassan A., Ahmad A. (2014). The Effects of Tualang Honey Supplement on Medial Prefrontal Cortex Morphology and Cholinergic System in Stressed Ovariectomised Rats.

[bib4] Al-Rahbi B., Zakaria R., Othman Z., Hassan A., Muthuraju S., Wan Mohammad W.M. (2013). Mood and memory function in ovariectomised rats exposed to social instability stress. BioMed Res. Int..

[bib6] Arnsten A.F., Wang M.J., Paspalas C.D. (2012). Neuromodulation of thought: flexibilities and vulnerabilities in prefrontal cortical network synapses. Neuron.

[bib7] Barch D.M., Ceaser A. (2012). Cognition in schizophrenia: core psychological and neural mechanisms. Trends Cognit. Sci..

[bib8] Camina E., Guell F. (2017). The neuroanatomical, neurophysiological and psychological basis of memory: current models and their origins. Front. Pharmacol..

[bib9] Cirillo M.A., Seidman L.J. (2003). Verbal declarative memory dysfunction in schizophrenia: from clinical assessment to genetics and brain mechanisms. Neuropsychol. Rev..

[bib10] Deiana S., Platt B., Riedel G. (2011). The cholinergic system and spatial learning. Behav. Brain Res..

[bib11] Doshi S.B., Agarwal A. (2013). The role of oxidative stress in menopause. J. Mid Life Health.

[bib12] Forbes N.F., Carrick L.A., McIntosh A.M., Lawrie S.M. (2009). Working memory in schizophrenia: a meta-analysis. Psychol. Med..

[bib13] Green M.F., Nuechterlein K.H., Gold J.M., Barch D.M., Cohen J., Essock S., Marder S.R. (2004). Approaching a consensus cognitive battery for clinical trials in schizophrenia: the NIMH-MATRICS conference to select cognitive domains and test criteria. Biol. Psychiatr..

[bib14] Hajjar I., Hayek S.S., Goldstein F.C., Martin G., Jones D.P., Quyyumi A. (2018). Oxidative stress predicts cognitive decline with aging in healthy adults: an observational study. J. Neuroinflammation.

[bib15] Hatanaka H., Hanyu H., Hirose D., Fukusawa R., Namioka N., Iwamoto T. (2015). Peripheral oxidative stress markers in individuals with Alzheimer's disease with or without cerebrovascular disease. J. Am. Geriatr. Soc..

[bib16] Kahn R.S., Keefe R.S. (2013). Schizophrenia is a cognitive illness: time for a change in focus. JAMA Psychiatry.

[bib17] Keefe R.S., Buchanan R.W., Marder S.R., Schooler N.R., Dugar A., Zivkov M., Stewart M. (2013). Clinical trials of potential cognitive-enhancing drugs in schizophrenia: what have we learned so far?. Schizophr. Bull..

[bib18] Keefe R.S.E., Fenton W.S. (2007). How should DSM-V criteria for schizophrenia include cognitive impairment?. Schizophr. Bull..

[bib19] Khalil M.I., Alam N., Moniruzzaman M., Sulaiman S.A., Gan S.H. (2011). Phenolic acid composition and antioxidant properties of Malaysian honeys. J. Food Sci..

[bib20] Khoo Y.T., Halim A.S., Singh K.K., Mohamad N.A. (2010). Wound contraction effects and antibacterial properties of Tualang honey on full-thickness burn wounds in rats in comparison to hydrofibre. BMC Compl. Alternative Med..

[bib21] Leal G., Afonso P.M., Salazar I.L., Duarte C.B. (2015). Regulation of hippocampal synaptic plasticity by BDNF. Brain Res..

[bib22] Lezak M.D., Howieson D.B., Loring D.W. (2004). Neuropsychological Assessment.

[bib38] Maj M., D'Elia L., Satz P. (1993). Evaluation of two new neuropsychological tests designed to minimize cultural bias in the assessment of HIV-1 seropositive persons: a WHO study. Arch. Clin. Neuropsychol..

[bib23] Malaysia M.O.H. (2005). Schizophrenia Report 2003- 2005.

[bib24] Moniruzzaman M., Sulaiman S.A., Khalil M.I., Gan S.H. (2013). Evaluation of physicochemical and antioxidant properties of sourwood and other Malaysian honeys: a comparison with manuka honey. Chem. Cent. J..

[bib25] Nasir N.A., Halim A.S., Singh K.K., Dorai A.A., Haneef M.N. (2010). Antibacterial properties of tualang honey and its effect in burn wound management: a comparative study. BMC Compl. Alternative Med..

[bib26] Nguyen H.T.L., Panyoyai N., Kasapis S., Pang E., Mantri N. (2019). Honey and its role in relieving multiple facets of atherosclerosis. Nutrients.

[bib27] Nurul Syazana M.S., Halim A.S., Gan S.H., Shamsuddin S. (2011). Antiproliferative effect of methanolic extraction of tualang honey on human keloid fibroblasts. BMC Compl. Alternative Med..

[bib28] Othman Z., Shafin N., Zakaria R., Hussain N.H., Mohammad W.M. (2011). Improvement in immediate memory after 16 weeks of tualang honey (Agro Mas) supplement in healthy postmenopausal women. Menopause.

[bib29] Overall J.E., Gorham D.R. (1962). The brief psychiatric rating scale. Psychol. Rep..

[bib30] Rey A. (1958). L'examen clinique en psychologie. [The clinical examination in psychology].

[bib31] Ruzita Jamaluddin Z.O., Musa K.I., Alwi M.N.M. (2009). Validation of the Malay version of auditory verbal learning test (mvavlt) among schizophrenia patients in hospital Universiti Sains Malaysia (Husm), Malaysia. ASEAN J. Psychiatry.

[bib32] Schliebs R., Arendt T. (2011). The cholinergic system in aging and neuronal degeneration. Behav. Brain Res..

[bib33] Schmidt M. (1996). Rey Auditory Verbal Learning Test: A Handbook.

[bib34] Shafin N., Othman Z., Zakaria R., Nik Hussain N.H. (2014). Tualang honey supplementation reduces blood oxidative stress levels/activities in postmenopausal women. ISRN Oxidative Medicine.

[bib35] Sinkeviciute I., Begemann M., Prikken M., Oranje B., Johnsen E., Lei W.U., Sommer I.E. (2018). Efficacy of different types of cognitive enhancers for patients with schizophrenia: a meta-analysis. NPJ schizophrenia.

[bib36] Siuda J., Patalong-Ogiewa M., Zmuda W., Targosz-Gajniak M., Niewiadomska E., Matuszek I., Rudzinska-Bar M. (2017). Cognitive impairment and BDNF serum levels. Neurol. Neurochir. Pol..

[bib37] WHO (2018). Schizophrenia. https://www.who.int/news-room/fact-sheets/detail/schizophrenia.

